# Efficacy and safety of ^225^Ac-PSMA-617 targeted alpha therapy in metastatic castration-resistant Prostate Cancer patients

**DOI:** 10.7150/thno.48107

**Published:** 2020-07-23

**Authors:** Madhav Prasad Yadav, Sanjana Ballal, Ranjit Kumar Sahoo, Madhavi Tripathi, Amlesh Seth, Chandrasekhar Bal

**Affiliations:** 1 Department of Nuclear Medicine, All India Institute of Medical Sciences, New Delhi, India.; 2 Department of Medical Oncology, BR Ambedkar Rotary Cancer Hospital, All India Institute of Medical Sciences, New Delhi, India.; 3 Department of Urology, All India Institute of Medical Sciences, New Delhi, India.

**Keywords:** ^225^Ac-PSMA-617 therapy, Targeted alpha therapy, mCRPC, efficacy, safety, salvage treatment

## Abstract

**Rationale:** Despite the success of several standards of care treatment options in metastatic castration-resistant prostate cancer (mCRPC), a significant number of patients attain therapeutic resistance and eventually develop disease progression. Managing these patients are currently challenging. Hence, there is an unmet need for further efficient therapeutic options that induce anti-tumor activity and improve survival. The objective of this study was to assess the safety and therapeutic efficacy of ^225^Ac-PSMA-617 targeted alpha therapy (TAT) in mCRPC patients in real-world conditions.

**Methods:** In this prospective study, we recruited patients with mCRPC who either were refractory to ^177^Lu-PSMA-617 radioligand therapy (RLT) or did not receive previous ^177^Lu-PSMA-617 RLT. Patients were treated with ^225^Ac-PSMA-617 TAT (100 KBq/Kg body weight) at 8-weekly intervals. The primary endpoint included the assessment of biochemical response by measuring the serum prostate-specific antigen (PSA) response rate as per the prostate cancer working group criteria (PCWG3). Secondary endpoints comprised the estimation of overall survival (OS), progression-free survival (PFS), molecular tumor response assessment (PERCIST 1 criteria), disease control rate (DCR), toxicity according to CTCAE v5.0, and clinical response evaluation.

**Results:** A total of 28 patients were recruited for this cohort study among whom 15 (54%) received prior ^177^Lu-PSMA-617 RLT and the remaining 13 (46%) patients were ^177^Lu-PSMA-617 RLT naïve. The mean age was 69.7 years (range: 46-87 years). All patients, except one, had extensive skeletal metastases on baseline ^68^Ga-PSMA-11 PET/CT scan; one patient had lymph node dominant disease and advanced primary prostatic tumor. The mean activity administered was 26.5 ± 12 MBq (range: 9.25 - 62.9 MBq) [715.5 ± 327 µCi, range: 250 - 1700 µCi] with a median of 3 cycles (range: 1 - 7 cycles). At 8^th^ week of post first cycle of ^225^Ac-PSMA-617 therapy (initial follow-up) and the end of the follow-up, >50% decline in PSA was observed in 25% and 39%, respectively. The median PFS and OS were 12 months (95% CI: 9 - 13 months) and 17 months (95% CI: 16 months - upper limit not reached), respectively. Molecular tumor response by PERCIST 1 criteria could be conducted in 22/28 (78.6%) patients, which revealed complete response in 2/22 (9%), partial response in 10/22 (45.4%) patients, 2/22 (9%) with stable disease, and 8/22 (36%) with progressive diseases. The disease control rate, according to the biochemical and molecular tumor response criteria, was 82% and 63.6%, respectively. Multivariate analysis revealed PSA progression as adverse prognostic indicator of OS, and any PSA decline as a good prognostic indicator of PFS. There was no Grade III/IV toxicity noted in this series. The most common side-effect was transient fatigue (50%) followed by grade I/II xerostomia (29%).

**Conclusion:** ^225^Ac-PSMA-617 TAT showed promising disease control rate, even when all other therapeutic options were exhausted, with low treatment-related toxicities.

## Introduction

In recent years, ^177^Lu-PSMA-617 radioligand therapy (RLT) has gained importance and has been widely accepted in the treatment of metastatic castration resistant prostate cancer patients (mCRPC) 1-5. If the results of the ongoing, international phase III VISION trial on ^177^Lu-PSMA-617 plus best standard of care versus only chemotherapy best standard of care in 750 patients prove positive, it may lead to the FDA approval (NCT03511664). A recent meta-analysis in mCRPC patients revealed that despite the promising results of ^177^Lu-PSMA-617 therapy, approximately 87/234 (37.1%; 95% CI, 33.9 - 40.3%) patients are non-responders and demonstrate clinical and/or biochemical prostate-specific antigen (PSA) progression 6.

The question does arise as to what is the next option if mCRPC patients are resistant to docetaxel/cabazitaxel, androgen deprivation therapies (ADTs), next-generation anti-androgens and even refractory to ^177^Lu-PSMA-617 beta-particle RLT? The superior radiobiological property of alpha radiation {Radiation Weighting Factor (Wr)-20} over beta radiation {Radiation Weighting Factor (Wr)-1} is an encouraging option to enhance the efficacy of RLT by radiolabelling the PSMA-617 ligand with alpha-particle emitters 7.

The anti-tumor effects of ^225^Ac-PSMA in animals 8, as well as initial studies in mCRPC patients, are published by a handful of investigators 9-12 who reported variable toxicities and safety data, albeit, the high objective response rates. The toxicity could be due to prior therapeutic modalities used in these patients or associated co-morbid conditions or racial differences that need careful investigations in different geographical locations to make any definite conclusion. The therapeutic efficacy of ^225^Ac-PSMA-617 targeted alpha therapy (TAT) by Kratochwil *et al.* 11 demonstrated sustained tumor-control in those patients, who have progressed despite standard therapeutic options and ^177^Lu-PSMA-RLT. Sathekge *et al.* 12 reported a decline of ≥90% PSA levels from the baseline in 82% (14/17) of advanced staged, prostate cancer patients; however, the patients included were chemotherapy-naïve. The patients who were treatment naïve and administered ^225^Ac-PSMA-617 as the first therapy option are likely to do better than those who were at the end of all approved therapy options. The former study is a more real-world situation than the latter published from the previous studies 11,12. Interestingly, initial therapeutic outcomes of ^225^Ac-PSMA-617 TAT from both the studies are encouraging, and if proved to be consistent in the future studies, this radiopharmaceutical may have an essential place in the field of prostate cancer management.

We have been treating mCRPC patients using ^177^Lu-PSMA-617 as a radioligand therapy, which has proved promising and have successfully treated 92 mCRPC patients 5. However, in view of the promising results of the pilot studies 9-12, we decided to offer ^225^Ac-PSMA-617 TAT as an option for heavily pre-treated mCRPC patients. This prospective cohort study aimed to report data on the safety and efficacy of ^225^Ac-PSMA-617 TAT in mCRPC patients.

## Materials and Methods

### Patients

In this prospective cohort study, mCRPC patients were screened from the Medical Oncology OPD of AIIMS and other tertiary care hospitals for the treatment with ^225^Ac-PSMA-617 TAT between April 2018 and January 2020, at the Department of Nuclear Medicine, AIIMS, New Delhi, India. The study was initiated following ethical approval from the human ethics committee [IEC-518/2018, RP-18/2018].

### Inclusion criteria

The eligibility criteria for ^225^Ac-PSMA-617 TAT included histological or cytologically confirmed prostatic adenocarcinoma patients with history of medical or surgical castration, patients who received prior second generation anti-androgen therapy (arbiraterone and enzalutamide), chemotherapy or categorized unfit for chemotherapy, patients refractory to ^177^Lu-PSMA-617 RLT or directly opted ^225^Ac-PSMA-617 TAT, patients on concomitant medications for the treatment of mCRPC, demonstration of intense PSMA expression on ^68^Ga-PSMA-11 PET/CT scan with uptake in the lesions greater or equal to the liver, patients with adequate bone marrow, liver and renal function: haemoglobin ≥ 8 g/dL, absolute neutrophil count (ANC) ≥1.5 x 10^9^ /L, Platelets >60 x 10^9^ /L, bilirubin ≤1.5 X upper normal limit (UNL), Adequate renal function {creatinine ≤1.6 mg/dL, GFR ≥40 mL/min/1.73m^2^ BSA (^99m^Tc-DTPA plasma sample method)}, patients with ECOG performance status ≤ 4. Any patient not fulfilling the above criteria or not willing to give written consent for the treatment was excluded from the study. After screening the patients with the above criteria, out of 31 consecutive mCRPC, 3 did not follow-up after the first cycle of ^225^Ac-PSMA-617 therapy and were hence excluded from the analysis. Finally, 28 patients with a mean age of 69.7 ± 9.4 (46 - 87) years were included for the analysis. Among the 28 patients, 15 (54%) were refractory to prior ^177^Lu-PSMA-617 RLT, and the remaining 13 (46%) patients directly received ^225^Ac-PSMA-617 therapy.

### ^68^Ga-PSMA-11 PET/CT image acquisition

Baseline pre-therapy diagnostic ^68^Ga-PSMA-11 PET/CT scans were obtained in all the 28 patients.^68^Ga-PSMA-11 PET/CT scans were acquired approximately 40 min after the intravenous injection of 74 - 185 MBq of ^68^Ga-PSMA-11. All the scans were performed on the Biograph mCT (Siemens, Erlangen, Germany) 64-slice PET/CT scanner. The acquisition parameters involved an initial topogram of the whole body, followed by a low dose CT from the vertex to mid-thigh. The 3D emission data were acquired at 2 min per bed position and corrected for random, scatter, and decay. Image reconstructions were carried out using iterative reconstruction (2 iterations, 21 subsets). All the studies were processed in the multi-modality work port (MMWP) Siemens processing system.

The follow-up ^68^Ga-PSMA-11 PET/CT scans were acquired in all, except 6 patients, due to logistic reasons (frequent travel constraints from far of places).

### Radiopharmaceutical Labelling

^225^AcCl_3_ was procured from ITG (Garching, Germany) and radiolabelled with PSMA-617 ligand, which was obtained from CMR (Center of Molecular Research, Moscow, Russia). A stock solution of ^225^AcCl_3_ in 0.01 M HCl was added to the sodium ascorbate buffer-PSMA-617 mixture and heated at 90 °C for 10 min. Quality control of radiolabelled products was performed using instant thin-layer chromatography, and the radiochemical purity was assessed by the method adopted by Kratochwil *et al.* 13.

### Treatment and Follow-up

**Figure 1:**
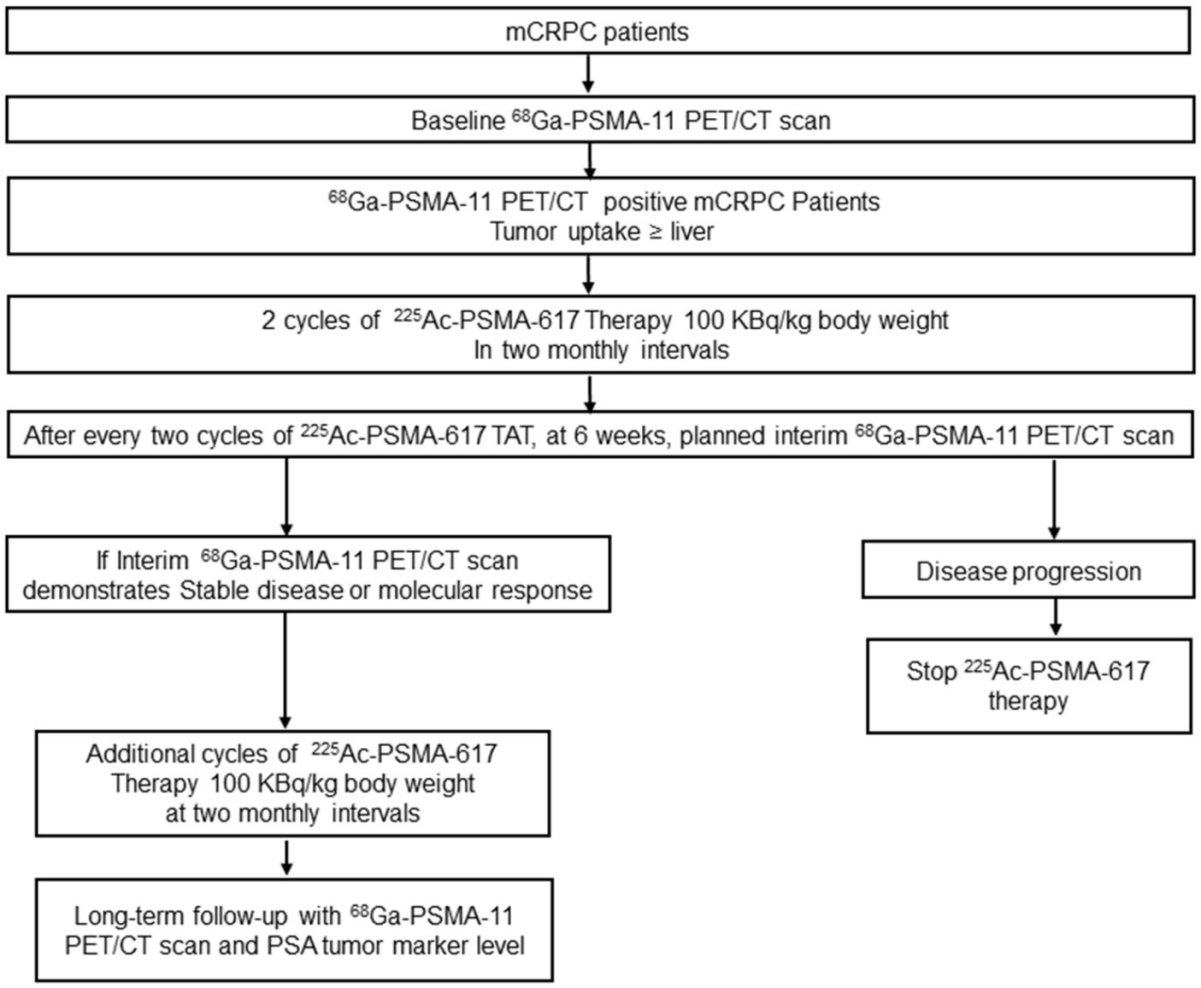
Flow chart depicting the treatment protocol and follow-up for ^225^Ac-PSMA-617 therapy.

The treatment protocol is detailed in Figure 1.

#### Patient Preparation

Before every cycle of ^225^Ac-PSMA-617 TAT, complete blood counts (CBC), kidney function tests (KFT), glomerular filtration rate (GFR), liver function tests (LFT), and serum prostate-specific antigen (PSA) levels were documented in all patients.

#### ^225^Ac-PSMA-617 Infusion

A fixed-dose of 100 KBq/kg body weight (BW) (2.7 µCi/Kg BW) of ^225^Ac-PSMA-617 was administered at 8-weekly intervals up to a cumulative dose of 62.9 MBq (1700 µCi) ranging between 9.25 to 62.9 MBq (250 - 1700 µCi). The Infusion involved a dilution of ^225^Ac-PSMA-617 in 30 mL normal saline (0.9%), which was administered intravenously over 5 - 10 min, followed by flushing with 20 mL normal saline. As a conservative approach, the entire process was performed in the isolation therapy ward, and patients were monitored for 24 h post-therapy for any acute side effects, even if there was no radiation regulatory requirement for alpha therapy.

#### Follow-up

As a routine follow-up, CBC, KFT, GFR, and LFT were assessed at 2, 4, and 8 weeks after each cycle of ^225^Ac-PSMA-617 TAT.

### Treatment Outcome Endpoints

The evaluation of the PSA response rate was the primary endpoint of this study. Secondary endpoints included progression-free survival (PFS), overall survival (OS), molecular tumor response assessment, disease control rate (DCR), adverse event profile, and clinical response assessment.

#### Biochemical Response

Biochemical response to treatment was assessed according to the Prostate Cancer Working Group 3 criteria (PCWG3). According to the criteria, partial response was a >50% decline in PSA from the baseline with confirmation after 3 - 4 weeks, and a >25% increase in PSA level above the baseline was deemed biochemical disease progression 14.

**PSA response rate**: defined as the percentage of patients with a PSA reduction of > 50% from baseline.

Timepoints of assessment: 2, 4, and 8 weeks after every cycle of ^225^Ac-PSMA-617 TAT. After completion of the required ^225^Ac-PSMA-617 cycles, PSA was repeated at monthly intervals.

#### Survival Analysis

**Overall survival:** defined as the time from the initiation of ^225^Ac-PSMA-617 therapy to the occurrence of death due to any cause or the date of the last contact.

**Progression-free survival:** defined as the time from the start of treatment date to the date of first observation of documented disease progression (PSA rise >25% from baseline value/molecular progression, whichever occurred first).

#### Molecular Tumor Response

The molecular response was assessed using PERCIST 1 criteria 15. According to the criteria, CR was a complete resolution of ^68^Ga-PSMA-11 uptake in the target lesions. Partial response (PR) was defined as >30% decrease in the SUL peak uptake of the target lesions from the baseline scan. Neither PR nor complete response (CR) nor progressive disease (PD) was considered a stable disease (SD). The progressive disease (PD) was defined as >30% increase in the SULpeak value of the target lesions from the baseline scan.

**The time points of assessment:** The assessments were performed at baseline before the initiation of ^225^Ac-PSMA-617 therapy and restaging of disease at 6 ± 1 week after every two cycles of ^225^Ac-PSMA-617 therapy.

#### Disease-control rate

The DCR is defined as the ratio of patients who have attained a response or stable disease to the total number of patients included in the study. In this study, DCR was calculated according to both biochemical and molecular imaging response.

#### Toxicity

Adverse events were recorded as per the National Cancer Institute for Common Toxicity Criteria version 5.0 16.

#### Clinical Response

Criteria such as visual analog score (VAS) 17, analgesic score (AS) 17, Karnofsky performance status (KPS) 18, and Eastern Cooperative Oncology Group (ECOG) performance status were parameters used to assess the clinical response.

Visual analogue scoring was used for pain documentation 17. The intensity of pain on scale ranged from 0 to 10, of which 0 scores implied no pain; score 6 implied moderate pain and 10 implied intolerable pain. On the basis of VAS scoring, the response was divided into 4 categories: complete response (>70% decrease in VAS), partial response (PR, 40 - 70% decrease in VAS), minimal response (MR, 20 - 40% decrease in VAS), and no response (<20 % decrease in VAS or increase in pain). Analgesic scoring was conducted as per the Urological Group of the European Organization of Research and Treatment of Cancer (EORTC, Protocol 30921). The analgesic score is the product of two five-point scales (the type of analgesic and the frequency of its administration). A decline in the analgesic score was documented as a response to treatment. A decrease in the analgesic scoring was considered a positive response to treatment. KPS and response criteria of the ECOG performance status were used to assess the quality of life. The KPS ranged from 100 to 0 (100: no evidence of disease and no complaints; 0: dead) 18. The ECOG status ranged from 0 to 5 (0: fully active, able to carry on all pre-disease performance without restriction and 5: dead).

### Statistics

The D'Agostino-Pearson test was conducted to assess the normality of the data. The normal distributed continuous data were presented as mean, standard deviation, and range. Skewed data were depicted as the median and interquartile range (IQR). In order to report detailed results, the patients treated with ^225^Ac-PSMA-617 TAT were categorized into two groups based on the prior ^177^Lu-PSMA-617 RLT status: Gr-I (N = 15); prior ^177^Lu-PSMA-617 RLT group, Gr-II (N = 13); ^177^Lu-PSMA-617 RLT naïve group. The patient groups were compared using unpaired samples t-test (parametric test) or Mann-Whitney test (non-parametric test). Paired-samples t-test (parametric) or Wilcoxon signed-rank test (non-parametric) tests were used to compare the pre and post-therapy parameters. Waterfall plots demonstrated the percentage change in the PSA levels from the baseline at different timepoints. Kaplan-Meier survival curves were generated, and the Log-rank test was used to compare the OS and PFS of categorized variables. Their median values designated the cut-offs for continuous variables. For multivariate analysis, the Cox proportional-hazards regression model by stepwise, backward elimination method was carried out to determine the prognostic factors associated with OS and PFS. The analysis was carried out with the MedCalc software. P-values <0.05 were considered significant.

## Results

### Patients

The treatment was administered between April 2018 and January 2020. Table 1 enumerates the details regarding the clinical history, prior anti-cancer medications, and ^225^Ac-PSMA-617 therapy dosage details of the patients included in the analysis.

All patients (96%), except one, demonstrated skeletal metastases. The lymph node metastases were prevalent in 85.7% of the patients, usually associated with bone metastases; 31.7% of patients had other metastasis which included, lung, liver, brain, and adrenal metastases (Table 1). Interestingly, 82% of patients had PSMA expressing disease at the primary site. All the patients had received at least two lines of prior treatment, and 85.7% (24/28) were treated with three or more lines (Table 1). Eight (28.5%) patients were on concomitant ^225^Ac-PSMA-617 plus anti-androgen/androgen inhibitor therapy. 28% and 72% of patients belonged to the ECOG performance status category ≤2 and ≥3, respectively.

In a median follow-up duration of 10 months (range: 5 - 22 months), the mean cumulative radioactivity administered was 26.5 ± 12 MBq (range: 9.25 - 62.9 MBq) [715.5 ± 327 µCi, range: 250 - 1700 µCi].

Out of a total of 85 cycles administered, 10 patients completed 2 cycles, 8 patients received 3 cycles, and 7 patients had 4 cycles of therapy. Among the remaining 3 patients: 1 patient received 5 cycles, 1 patient had even 7 cycles, and one patient received only 1 cycle of ^225^Ac-PSMA-617 TAT because he died of viral meningitis before the second cycle.

To assess if the prior administration of ^177^Lu-PSMA-617 RLT had any bearing on the outcome/toxicity of subsequent therapy with ^225^Ac-PSMA-617; we did the post-hoc analysis. Out of 28 patients included in the study, 15 (54%) were refractory to ^177^Lu-PSMA-617 (Group I; [Gr-I]), and the remaining 13 (46%) patients did not receive prior ^177^Lu-PSMA-617 therapy (Group-II; [Gr-II]). The baseline characteristics of both the patient groups are detailed in table 2. Though patients were not randomized prospectively, both groups matched in terms of age, the extent of disease, and the cumulative administered activity of ^225^Ac-PSMA-617 (Table 2).

### PSA Response

#### PSA Response After First ^225^Ac-PSMA-617 TAT Cycle

**Figure 2:**
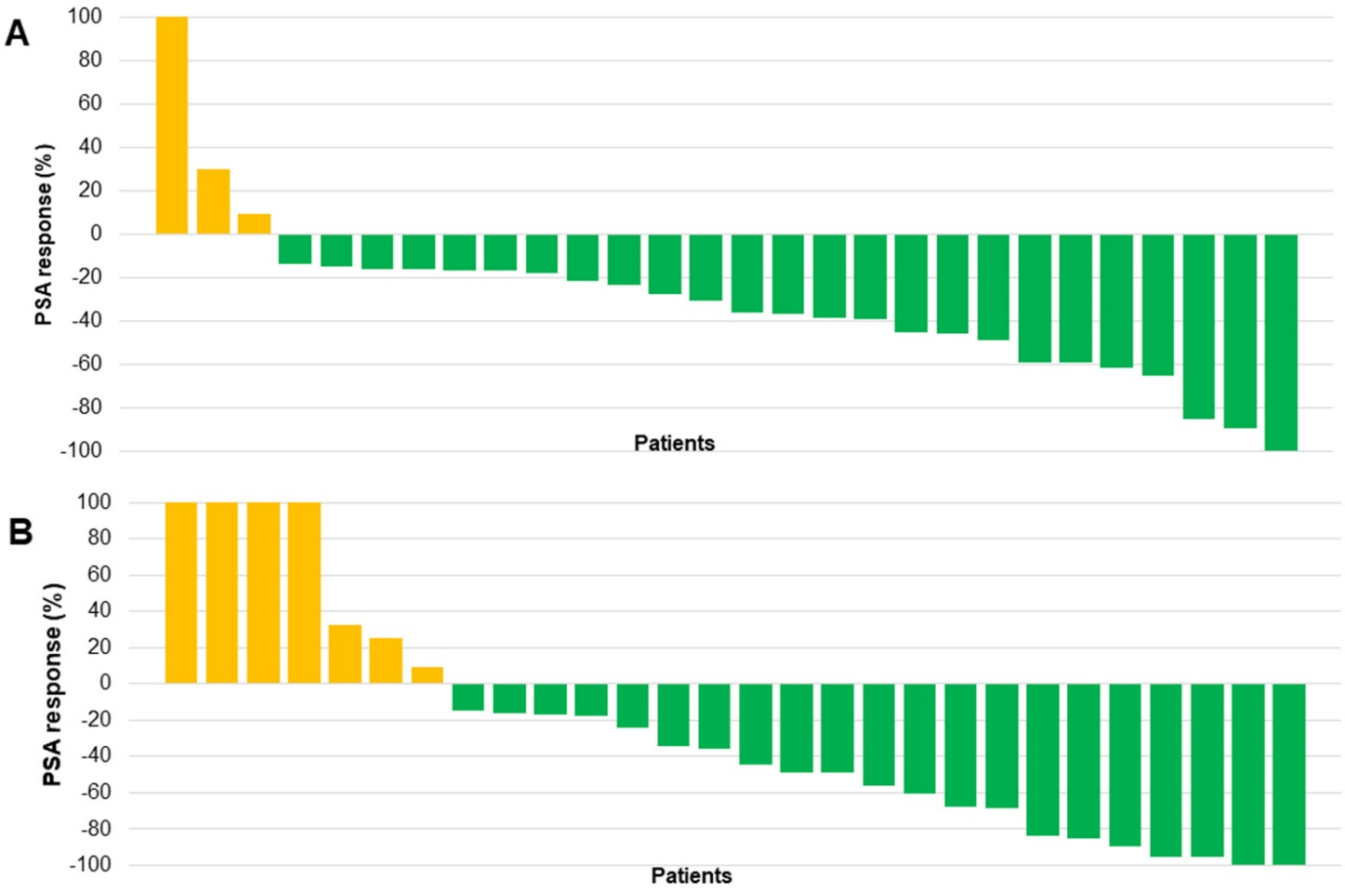
(**A**) Waterfall plots depicting percentage change in PSA response post-first cycle ^225^Ac-PSMA-617 TAT. (**B**) Waterfall plots depicting percentage change in PSA response at the end of follow-up after ^225^Ac-PSMA-617 TAT. *(the orange clour respresents patients with any PSA increase and the green colour depicts patients who experienced any percent PSA decline)*.

After the initial cycle of ^225^Ac-PSMA-617 TAT at a median of 8 weeks, 25/28 (89%) patients experienced any PSA decline; interestingly, 7 (25%) patients experienced remarkable >50% PSA decline even after the first cycle of TAT. However, 2/28 (7%) patients had a >25% increase in the PSA levels compared to the baseline serum PSA value. The percentage change in PSA response post-first cycle of ^225^Ac-PSMA-617 TAT for each patient is depicted in the form of a waterfall plot. The cut-off percentage was taken as ± 100% (Figure 2A).

#### PSA Response at the End Follow-up

At the time of end follow-up assessment, 22/28 (78.6%) patients exhibited any PSA decline. Among them, 11/28 (39%) patients demonstrated a PSA decline greater than 50%. Biochemical disease progression was observed in 5/28 (18%) patients, and in 1/28 (3.6%) patients, the serum PSA remained stable. The biochemical DCR was 23/28 (82%) and the percentage change in PSA at the last follow-up assessment is demonstrated in Figure 2B.

**Figure 3:**
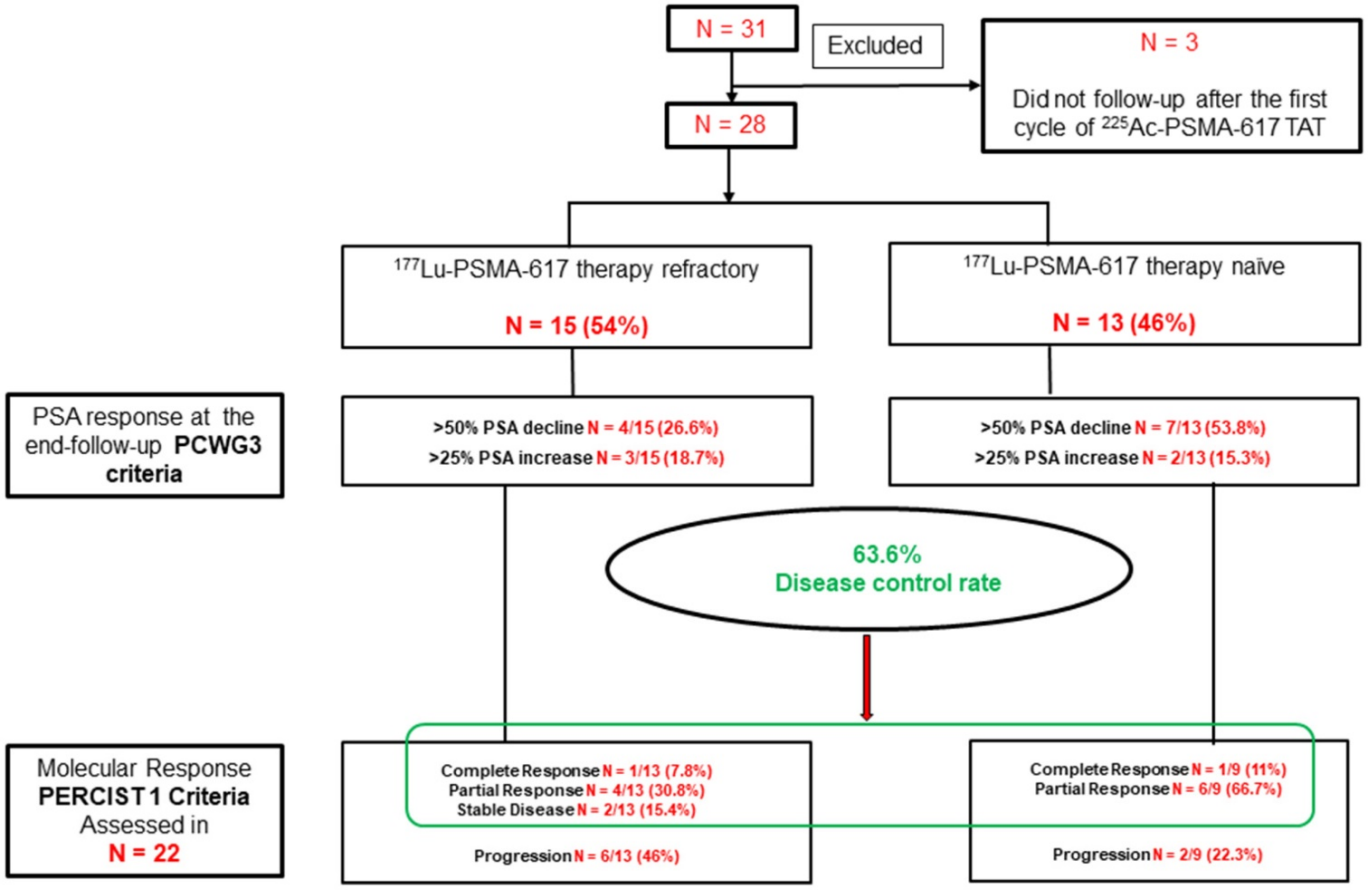
Flow chart depicting the biochemical and molecular imaging response rates in the prior ^177^Lu-PSMA-617 RLT refractory group (Gr-I) and naïve groups (Gr-II).

On subgroup analysis, when comparing the PSA response according to the status of prior ^177^Lu-PSMA-617 RLT, patients with no prior exposure to pervious ^177^Lu-PSMA-617 therapy demonstrated a significant PSA decline of >50% in 53.8% compared to only 26.6% in patients who received prior ^177^Lu-PSMA-617 therapy. However, both groups showed similar trends in the PSA progression (Figure 3).

An overall increase in the median PSA value was noted post-^225^Ac-PSMA-617 therapy in patients with progressive disease on prior ^177^Lu-PSMA-617 therapy [pre-therapy PSA: median 221.5 (IQR: 55.6 - 526.3 ng/mL) versus post-therapy PSA: median 234 (IQR: 278 - 512 ng/mL): P<0.001]. However, a significant PSA decline was observed in Group II patients [pretherapy PSA: median 282.4 (IQR: 28.6 - 386.5 ng/mL) versus post-therapy PSA: median 85.2 (IQR: 8.6 - 146.3 ng/mL)].

### Survival

**Figure 4:**
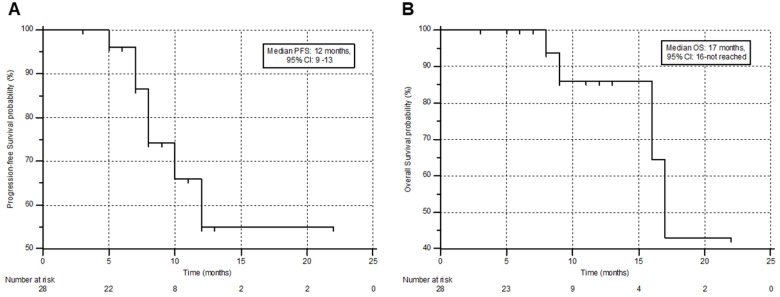
Kaplan-Meier plots depicting the progression-free survival (**A**) and overall survival (**B**) of the patients in months.

**Figure 5:**
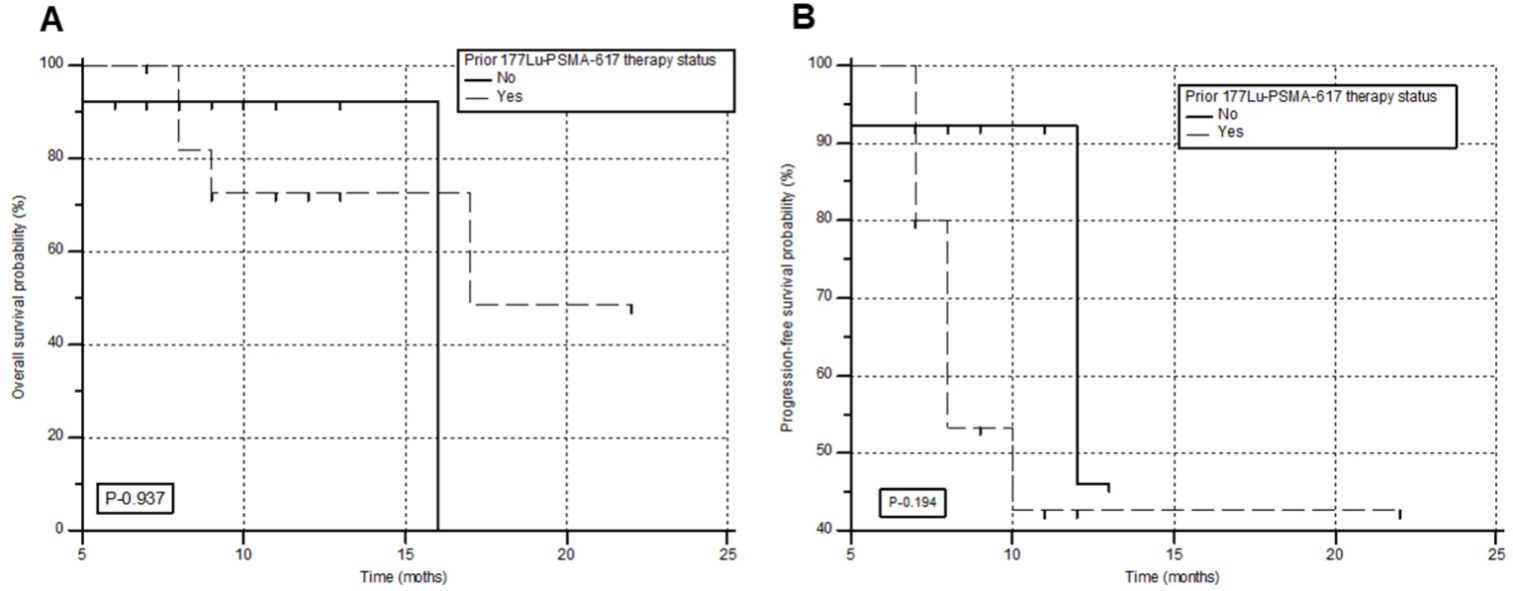
Overall survival (**A**) and progression-free survival (**B**) in months according to the prior status on ^177^Lu-PSMA-617 RLT.

During the follow-up, 6/28 (21.4%) patients died, among whom 4 patients had received prior ^177^Lu-PSMA-617 RLT. The median PFS and OS were 12 months (95% CI: 9 - 13 months) and 17 months (95% CI: 16 months - upper limit not reached), with a 12-month PFS and OS probability of 47.6% and 85.9 %, respectively. This information is shown in Figure 4A & B. A comprehensive, subgroup analysis according to the prior ^177^Lu-PSMA-617 RLT status, revealed no difference in the median PFS (Gr-I; 10 versus Gr-II; 12 months, P-0.194) and OS (Gr-I; 16 versus Gr-II; 17 months, P-0.937) between the groups (Figure 5A & B).

### Prognostic factors for survival

**Figure 6:**
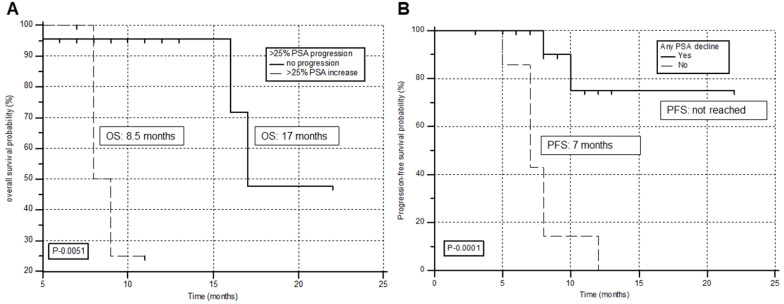
Kaplan-Meier survival curves of prostate cancer patients treated with^ 225^Ac-PSMA-617 TAT stratified by prognostic factors on multivariate analysis. (**A**) >25% PSA increase associated with overall survival. (**B**) Any PSA decline associated with progression-free survival.

The prognostic factors associated with OS and PFS are detailed in Supplementary Tables 1 & 2, respectively. Univariate analysis demonstrated tumor markers including, any PSA decline (P-0.017), > 50% PSA decline (P-0.022), and >25% PSA progression (P-0.0051) associated with the OS. However, multivariate analysis revealed only PSA increase >25% from the baseline as the prognostic factor associated with poor overall survival (hazard ratio [HR], 12.2; P = 0.033; 95% CI, 1.276 - 118). A significantly reduced estimated OS was observed for patients who demonstrated >25% PSA progression. (8.5 months for >25% PSA increase versus 17 months for patients with no PSA increase) (Figure 6A).

Similarly, from the univariate analysis reported any PSA decline (P-0.0001), >50% PSA decline (P-0.019), and >25% PSA progression (P-0.0001) associated with the PFS. However, from multivariate analysis, the only patient group who failed to show any PSA decline with^ 225^Ac-PSMA-617 TAT demonstrated the worst prognosis and were significantly associated with an inferior PFS (HR, 11.2; P-0.002; 95% CI, 2.333 - 54.632). A significantly inferior PFS was documented for patients who failed to show any PSA decline during the treatment period (median 7 months for no PSA decline versus not attained median PFS for patients experiencing any PSA decline) (Figure 6B).

### Molecular Response

The interim ^68^Ga-PSMA-11 PET/CT scan demonstrated 9% patients (2/22) attained complete molecular responses,a partial response in 45.4 % (10/22) patients, and stable disease in 9% (2/22) patients. The disease control rate (DCR), according to the molecular tumor response criteria, was 63.6% (14/22). On subgroup analysis, 5/13 (38.5%) versus 7/9 (78%) patients achieved a molecular response of disease in the prior ^177^Lu-PSMA-617 treatment group (Gr-I) versus ^177^Lu-PSMA-617 RLT naïve group (Gr-II), respectively. However, the difference in response was marginally significant (P-0.061), probably due to a small number of patients in each subgroup. During the follow-up, on molecular-basis disease progression was documented in 8/22 (36%) patients, interestingly, 6 of 8 patients (75%) had previously documented ^177^Lu-PSMA-617 RLT refractory disease (Figure 3).

### Adverse Events

The most common side effect from the TAT therapy in our series was transient grade I/II fatigue in 50% (14/28), followed by dry mouth in 29% (8/28) of patients who experienced only grade I/II toxicity. Among the 8 patients with dry mouth, 3 experienced grade I, and the remaining 5 patients showed grade II toxicity. Grade III fatigue was experienced in only one patient. Grade III anemia was reported in one patient. No evidence of grade III/IV thrombocytopenia, leukopenia, and hepatotoxicity was documented in the patients. A remarkable improvement in the number of patients with baseline grade III ALP was noted post-RLT (6 Vs. 3, P<0.001). Nephrotoxicity was limited to grade I only (Supplementary Table 3). One patient with ECOG status 4 at the baseline, received 1 cycle of ^225^Ac-PSMA-617 TAT and was due for his second cycle, but suffered from viral meningitis for which he was admitted, but died from the same.

### Clinical Response

When assessed clinically, post-treatment, there was a significant decrease in the VAS and the analgesic score, with a concordant improvement in the KPS and ECOG status from the baseline values (Table 3).

## Discussion

This prospective cohort study reports the efficacy and safety data on ^225^Ac-PSMA-617 therapy, which is a promising salvage treatment option for end-stage mCRPC patients.

Though the current study includes a moderate number of patients, the fair quality of the methodology and planned prospective design is the key strength of this study**.** This study comprehensively conducted a head-to-head comparison on the efficacy of ^225^Ac-PSMA-617 TAT between the ^177^Lu-PSMA-617 refractory and naïve group of patients. Whilst, it is not an ideal comparison as it is not a prospective RCT, the results of this pilot study shall aid in the designing of future large scale, two-armed RCT and provide information for sample size calculation based on effect size from this study.

We acknowledge the fact the study has a wide window of inclusion criteria which allows a heterogenous population of patients to be included in the study. The inclusion criteria were designed based on the idea of exploiting ^225^Ac-PSMA-617 therapy as a salvage treatment option. Hence, the broad spectrum of criteria were used such that patients even with a poor ECOG status up to 4 (Table 4), and patients who have exhausted all standard line of treatment options for this study could be included.

This study was conducted keeping in mind the use of ^225^Ac-PSMA-617 in the real life clinical contexts where worst of the worst cases are encountered by the Nuclear Medicine physician on daily basis. Unlike the previous reports with majority of the patient with ECOG ≤2 9 - 13 (Table 4), our patient population included 20/28 (72%) patients with ECOG 3 - 4 at the time of recruitment. Moreover, the study was designed based on practical situations, where patients usually attain an ECOG status 3/4 after heavy pre-treatmet with systemic therapies. Moreover, the number of publications on ^225^Ac-PSMA-617 are handful 11,12,19 and there are no phase III trial data available, hence any safety and efficacy data from different populations and settings will add to literature of ^225^Ac-PSMA-617 therapy in mCRPC patients.

On post hoc analysis, patients were divided wherein their detailed clinical history and response rates were stratified in two groups (Gr-I = 15 and Gr-II = 13), based on the prior treatment status with ^177^Lu-PSMA-617 therapy. Though it is not an ideal way to conduct a head-to-head comparison between the subgroups, interestingly, the baseline characteristics were matched, and hence, outcome measures were comparable.

Our results of 9% (2/22) complete response rate (CR) concerning PSA and ^68^Ga-PSMA-11 PET/CT was similar to 13% (5/38) CR as reported by Kratochwil *et al.* 11, however, Sathekge *et al.* 12 in their earlier publication reported significantly high CR of 64.7% (11/17). All 17 patients were chemotherapy-naive at the time of ^225^Ac-PSMA-617 TAT; this could partly explain the exceptionally high complete resolution of the metastases. Our hypothesis is furthermore validated by their recent publication of multicentric study, who were similar to our study recruits, exhausted all approved therapy options, were administered ^225^Ac-PSMA-617 TAT 19. The complete remission rate dropped to 29% and reported a progression rate of 31.5%.

Significantly higher biochemical response and marginally higher tumor response rates were observed in our patients who did not receive prior ^177^Lu-PSMA-617 RLT as the other salvage treatment option. Similarly, Sathekge *et al*. reported that the previous ^177^Lu-PSMA-617 therapy was negatively associated with PFS in multivariate analysis 19. Patients who have received previous exposure to ^177^Lu-PSMA-617 RLT (Gr-I) have exhibited a minor trend of lower disease control rate, and lower survival rate compared to the naïve group (Gr-II). However, they were not significantly associated with the OS and PFS (Supplementary Tables 1 & 2), (Figure 5A & B). One may argue that the radiation resistance acquired in the ^177^Lu-PSMA-617 RLT refractory patients might probably render ^225^Ac-PSMA-617 TAT less effective.

Kratochwil *et al.* 11 observed a higher rate of >50% PSA decline in 63% patients compared to only 39% in our patient group. Possible angles of eplanation for this discordance is that unlike our patient group where 72% of patients belonged to the ECOG performance status 3-4 category, Kratochwil *et al*. 11 and sathekge *et al's.* 19 patient cohort involved 20% and 18% of patients with ECOG status >2 (Table 4). Another possible explanation to the low PSA response trends in the ^177^Lu-PSMA-617 therapy refractory group patients may be the down-regulation of PSMA expression in the prostate cancer cells seen as diffuse or low uptake on ^68^Ga-PSMA-PET/CT scan 20. Further modifications of the treatment protocol with regards to the use of adjuvant radiosensitizers and dynamic escalation of ^225^Ac-PSMA-617 therapy dosage to 150 or 200 KBq/Kg BW may be the alternative options in these set of patients an initial tumor response to treatment, to achieve best disease control rate with minimal toxicity. However, these approaches were out of the scope of this study; in the future, this may prove prophetic.

**Figure 7:**
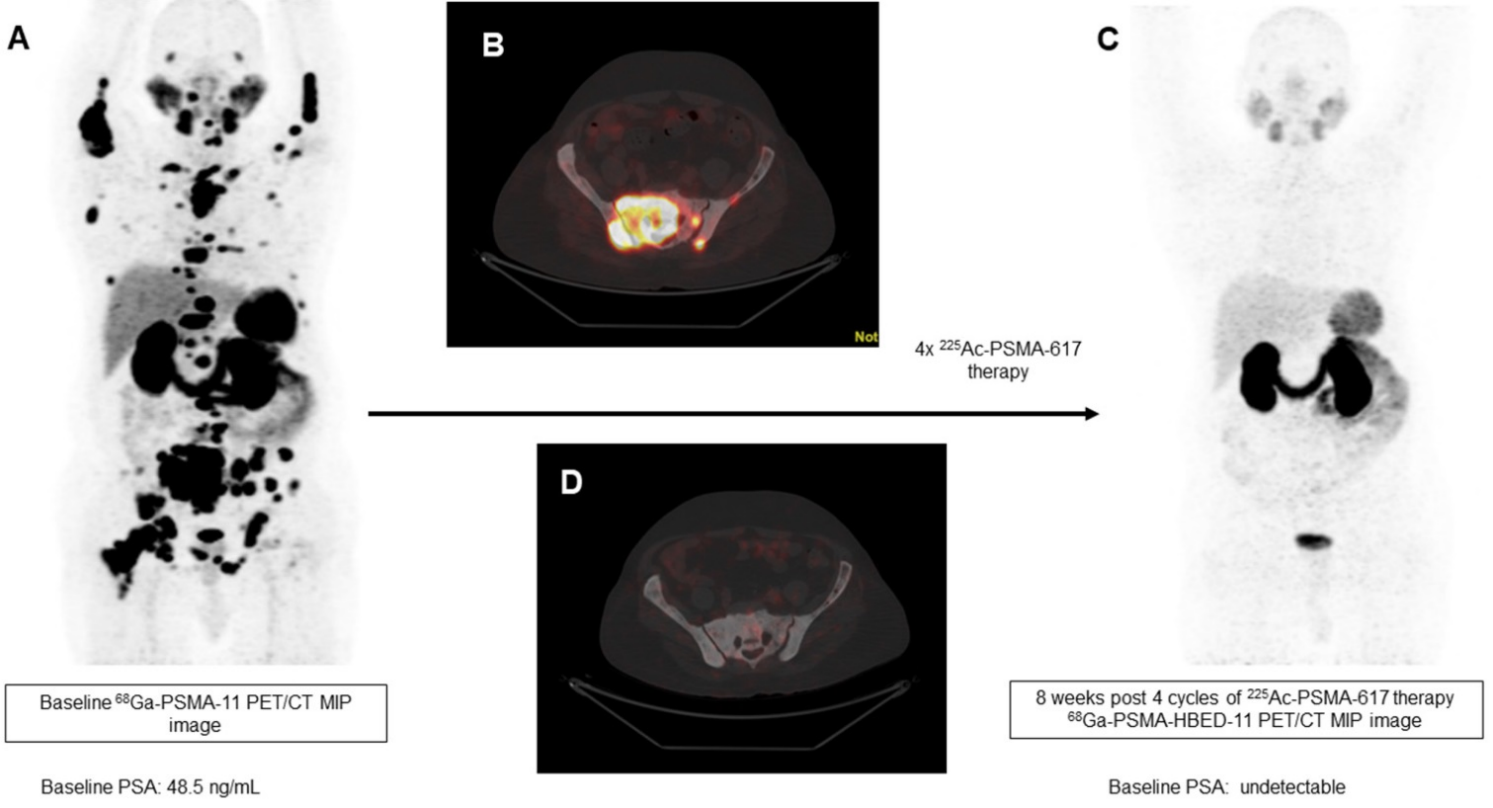
A 79-year-old prostate cancer patient treated with hormonal and chemotherapy and refractory to prior ^177^Lu-PSMA-617 presented with radiotracer-avid primary, lymph node, and extensive skeletal metastasis on pretherapy diagnostic ^68^Ga-PSMA-11 PET/CT scan (**A, B**). After the 4^th^ cycle of ^225^Ac-PSMA-617 therapy, the interim ^68^Ga-PSMA-11 PET/CT scan demonstrated complete resolution of the lesions (**C, D**) consistent with the complete molecular response.

**Figure 8:**
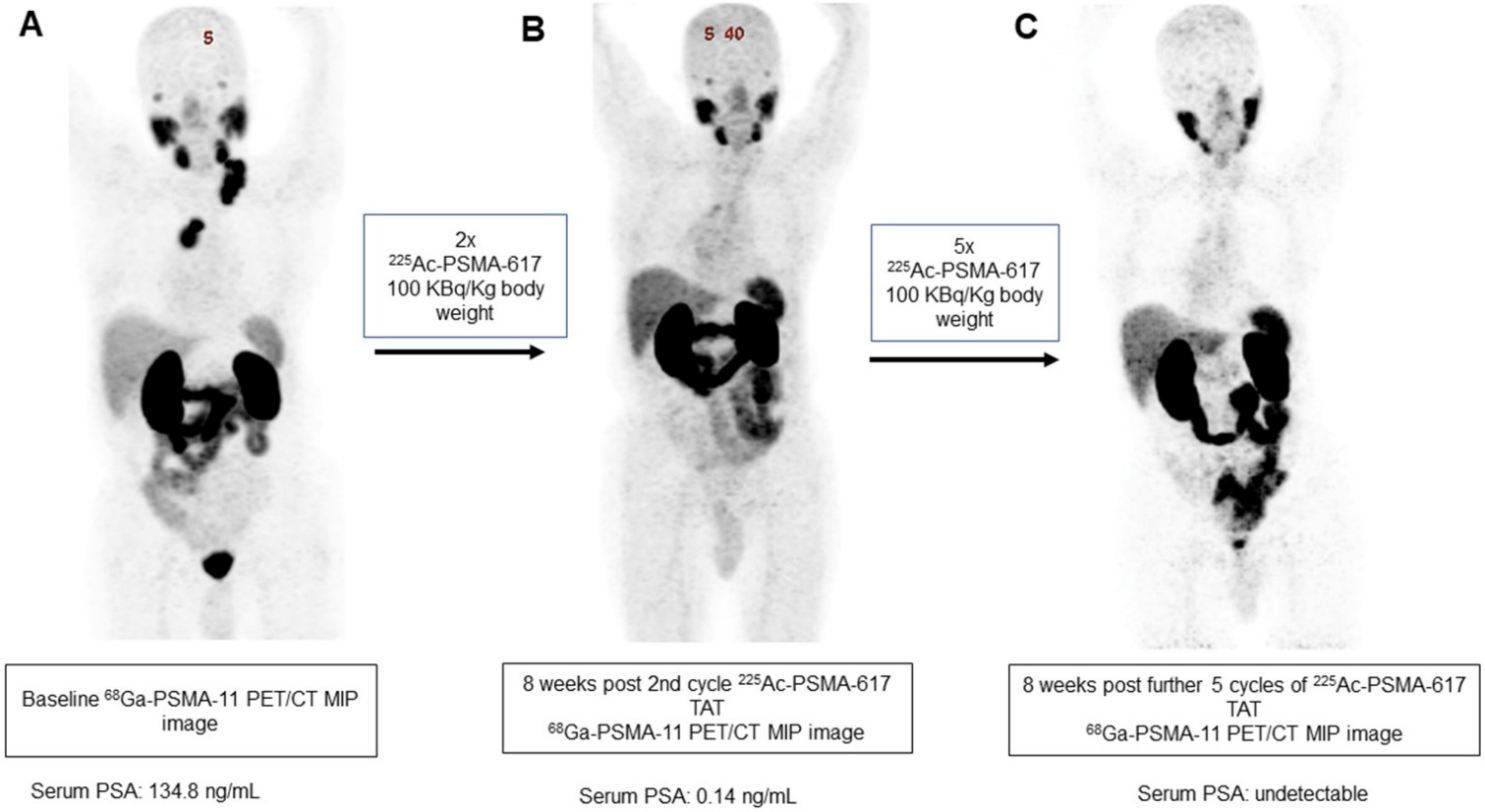
A 84-year-old prostate cancer patient treated with hormonal and chemotherapy presented with radiotracer-avid primary disease and lymph node metastasis on pretherapy diagnostic ^68^Ga-PSMA-11 PET/CT scan (**A**). After the 2^nd^ cycle of ^225^Ac-PSMA-617 therapy, the restaging ^68^Ga-PSMA-11 PET/CT scan revealed a remarkable decrease in the uptake, size, and number of all lesions except for minimal residual disease in mediastinal lymph nodes (**B**). After further 5 cycles of ^225^Ac-PSMA-617 TAT, ^68^Ga-PSMA-11 PET/CT scan demonstrated complete resolution of the lesions (**C**) consistent with the complete molecular response.

In our patient series, 2 patients achieved clinical, biochemical, and molecular complete remission. In the first patient, a remarkable complete resolution of disease was noted, who was heavily pretreated with standard therapies including ^177^Lu-PSMA-617 RLT. After receiving 4 cycles of fixed-dose 100 KBq/KgBW ^225^Ac-PSMA-617 therapy, no grade III/IV toxicities were documented, but the patient experienced fatigue and dry mouth as the main side-effects (Figure 7). The complete molecular and biochemical response was observed in the second patient, in whom the disease was limited to the primary and lymph nodes (Figure 8). This patient received a fixed dose of 100 KBq/KgBW/cycle for 7 cycles. After first two initial cycles of ^225^Ac-PSMA-617 therapy, ^68^Ga-PSMA PET/CT scan demonstrated a significant reduction in the cancer burden, revealed residual disease in the mediastinal lymph nodes with fluctuating PSA, hence, further 5 cycles were required to achieve complete biochemical, molecular and morphological response. During the entire treatment course and the follow-up duration, hematological, kidney, and hepatotoxicity were limited to grade I/II. The patient experienced moderate fatigue, which was transient and reduced approximately after 1 week of each cycle of ^225^Ac-PSMA-617 TAT. The onset of dry mouth symptoms occurred after the 2^nd^ cycle of therapy, which was persistent throughout the follow-up and was grade I/II. At the time of manuscript writing, both the patients are surviving and in complete remission.

Interestingly, among 36% of the patients who demonstrated molecular tumor progression, 75% were pretreated with and were refractory to ^177^Lu-PSMA-617 RLT. In this set of patients who had poor prognostic indicators and reached the end for all treatment options, ^225^Ac-PSMA-617 therapy improved the median time to progression and median overall survival time to 7 and 8.5 months, respectively. In patients with relentless progressive disease on ^177^Lu-PSMA-617 therapy, after alpha therapy, the biochemical (67%, 10/15) and molecular (47%, 7/15) disease control rates were remarkable.

Comparing our survival data with the historical reports, ^177^Lu-PSMA-617 therapy 6 demonstrated a PFS/OS of 11/13.7 months compared to 12/17 months with ^225^Ac-PSMA-617 therapy in a post-^177^Lu-PSMA-617 RLT setting in the current study. Interestingly, in line with our results, Kratochwil *et al.* 11 had reported PFS and OS of 7 and >12 months, respectively, in mCRPC patients (Table 4). This indicates that irrespective of the difference in PSA response rates, ^225^Ac-PSMA-617 demonstates a promising overall and progression-free susrvival benefit even given as the last-line treatment option. Despite the moderate response in the ^177^Lu-PSMA-617 RLT refractory group compared to the naïve group, the similar OS (16 vs 17 months, P-0.937) and PFS (10 vs 12 months, P-0.194) validates the fact that ^225^Ac interrupts the resistance of cancer cells to the beta emitting therapy (^177^Lu-PSMA-617 RLT) and initiates response in the refractory patients. A similar finding was observed by Sathekge *et al*. 19 who reported that the estimated PFS was much shorter in patients who received ^177^Lu-PSMA therapy prior to Ac-PSMA-617 therapy (5.1 months) compared with patients who did not (16.5 months).

Regarding identifying prognostic factors in the advanced mCRPC patients, Sathekge *et al*. 19 reported >50% PSA decline to govern the OS and PFS on multivariate analysis, as a favorable prognostic factor, however, we observed that even any PSA decline as the favorable prognostic indicators of OS and PFS and >25% PSA progression as a bad prognostic factor. Larger sample sizes with prolonged follow-up durations will be required to validate these findings.

In our study, a fixed ^225^Ac-PSMA-617 activity dosage of 100 kBq/kg (2.7 μCi/Kg) was adopted based on the reports of Kratochwil *et al*. 9, who extrapolated the previously established organ-based absorbed dose data on ^177^Lu-PSMA-617 to derive the absorbed doses of ^225^Ac-PSMA-617. The authors suggested the administered activity of 100 kBq/kg body weight of ^225^Ac-PSMA-617 as a reasonable balance between toxicity and therapeutic efficacy. We adopted the same therapeutic regimen, as suggested by Kratochwil *et al.* 9,11.

^225^Ac-PSMA-617 TAT was tolerated well, safe, and was confined majorly to low-grade toxicities. Only one patient experienced grade III/IV hematotoxicity, where the hemoglobin levels decrease from 9.6 to 7.4 g/dL. A drop in the hemoglobin and platelet count was observed from the baseline values, but was within grade I/II limit.

As 96% of the patients included presented with bone metastases, the underlying reason for bone marrow toxicity maybe radiation induced or inherent pancytopenia caused due to progression of bone metastases, however, the exact major contributor to the toxicity can be answered by microdosimetry techniques, which was out of scope of this paper. Similarly, no significant kidney toxicities were observed. However, the major clinical side effects of the therapy were fatigue, which was prevalent in 50%, followed by, dry mouth (29%). In agreement with our results, it is observed that xerostomia became the most common side effect when the treatment dose of ^225^Ac-PSMA-617 surpassed 100 kBq/kg per cycle 9. Unlike our results, Sathekge *et al.* 19 observed a remarkably high percentage (80%) of xerostomia, albeit low grade and tolerable, in their patient population, however, none warranted stopping RLT cycles. The reason could probably be that of ethnicity, but the actual reason is not yet understood. Deeper insight will be acquired by performing quantitative salivary gland scintigraphy studies to study the actual impairment in the salivary gland function.

Nine patients in the series received ≥ 4 cycles of ^225^Ac-PSMA-617 therapy, with a mean cumulative activity of 38 MBq (range 23-62.9 MBq) [1024.5 μCi (range 620-1700 μCi)]. Despite the higher cumulative doses administered, the toxicities were restricted to grade I/II. Prospective, dose-escalation studies, and its comparison with therapeutic efficacy, regular assessment of the adverse event, and long-term outcome data would aid in creating a balance between achieving the best therapeutic efficacy with minimal toxicities.

A majority of the patients have experienced a remarkable decrease in pain and show visual improvement in the KPS scoring on physical examination post-^225^Ac-PSMA-617 therapy. The maximum tolerable dose of ^225^Ac-PSMA-617 has not been derived about the non-availability of appropriate dosimetric tools, but, currently, at our clinic, we administered a maximum cumulative dose of 62.9 MBq and have not observed any serious adverse event related to therapy. ^225^Ac-PSMA-617 TAT approach in tandem 21 or post-^177^Lu-PSMA-617 RLT 11 or chemotherapy naïve patients 12, has proved beneficial in rapidly progressing symptomatic mCRPC patients.

## Limitations

The primary limitation of this study is the sample size. The purpose of sharing our data is to report very encouraging outcome measures even with a reasonable number of patients compared to the current literature. Secondly, post hoc analysis of the two groups of patients who were not randomized instead divided based on the previous ^177^Lu-PSMA-617 treatment. But the lead gives a significant clue for future planning of randomized control trials. Thirdly, the study could be considered as an interim result as it was analyzed at a shorter follow-up duration. Fourthly, the molecular tumor response was performed in 22 patients instead of all 28 patients.

## Conclusion

^225^Ac-PSMA-617 TAT is an effective salvage treatment option demonstrating high response rates with a reasonable trade-off with low treatment-related toxicities. It opens up a new vista for the patients who are refractory or relapsed from the standard line of treatments, including ^177^Lu-PSMA-617 therapy. ^225^Ac-PSMA-617 therapy induces moderate remission in ^177^Lu-PSMA-617 radioresistant mCRPC patients and improves survival. A multicentric randomized clinical trial is due to prove/disprove the initial cohort study outcomes.

## Supplementary Material

Supplementary tables.

## Figures and Tables

**Table 1:** Demographic characteristics of patients

Parameters	Values (N=28)
Age in years mean ± SD, (range),	69.7 ± 9.4 (46 - 87)
**Gleason score**	
7	6 (21.5%)
8	7 (25%)
9-10	15 (53.5%)
**Primary Treatment**	
Radical Prostectomy	3 (11%)
External beam radiotherapy to pelvis	7 (25%)
**Androgen deprivation therapy (ADT)**	
Surgical castration	15 (54%)
Medical castration ( LHRH agonist/antagonist)	13 (%)
**Anti-androgen therapy**	
First-generation	18 (64.2%)
**Treatment after attaining castration resistance**	
Second-generation anti-androgen (Enzalutamide)	10 (36%)
**Androgen synthesis inhibitor (Abiraterone acetate)**	22 (79%)
**Chemotherapy**	24 (93%)
Docetaxel	19 (79%)
Docetaxel + Cabazitaxel	4 (15%)
Cabazitaxel	1 (4%)
Median duration of androgen-deprivation therapy in months (IQR)	15 (12 - 21)
Median duration of anti-androgen therapy in months (IQR)	16 (11 - 26)
Median duration of androgen synthesis inhibitor therapy in months (IQR)	13 (10.7 - 14)
Median duration of chemotherapy in months (IQR)	11 (6 - 28)
Bisphosphonates	27 (96.4%)
External beam radiotherapy	12 (43%)
^177^Lu-PSMA-617 radioligand therapy	15 (54%)
Median number of ^177^Lu-PSMA-617 therapy cycles	3 (1 - 7)
Mean cumulative Activity of ^177^Lu-PSMA-617 TAT (MBq) ± SD, (range)	21 GBq (range, 1.11 - 34.780 GBq)
***Site and extent of disease on ^68^Ga-PSMA-11 PET/CT***	
Primary	23 (82%)
**Lymph nodes**	
Iliac and abdominal	14 (50%)
Thoracic to iliac	10 (35.7%)
No lymph nodes	4 (14.3%)
**Bone metastases**	
≤ 6	2 (7%)
6 - 20	11 (39%)
>20	14 (50%)
No bone metastases	1 (4%)
Diffuse/super scan/ Extensive	14 (50%)
Lung metastases	3 (10.7%)
**Other sites**	
Brain	2 (7%)
Liver	3 (10.7%)
Adrenals	1 (4 %)
Baseline median PSA (ng/mL) (median, 25 - 75% IQR)	222.2 (47 - 443.2)
Concomitant ati-androgen/androgen inhibitor therapy + ^225^Ac-PSMA-617 TAT	8 (29%)
Median follow-up after ^225^Ac-PSMA-617 therapy initiation in months (range)	10 (5 - 22)
Median number of ^225^Ac-PSMA-617 therapy cycles (range)	3 (1 - 7)
Mean cumulative Activity of ^225^Ac-PSMA-617 TAT (MBq) ± SD, (range)	26.5 ± 12 MBq (9.25 - 62.9 MBq)

ADT: Androgen deprivation therapy; AA: Abiraterone acetate; IQR: Inter quartile range; TAT: Targeted alpha therapy; PSA: Prostate specific antigen; SD Standard deviation.

**Table 2:** Baseline demographic characteristics of patients based on prior treatment status with ^177^Lu-PSMA-617 therapy

Variables	Prior ^177^Lu-PSMA-617 therapy Group (Gr-I) (N=15)	Prior ^177^Lu-PSMA-617 therapy Naïve Group (Gr-II) (N=13)	P-Value
Age (mean ± SD)	69.5 ± 9.8	70 ± 9.2	0.899
Gleason Score (Median)	9	9	0.980
**Prior Treatments**			
Surgery	12	6	0.926
First-generation Hormonal therapy	16	10	
Next-generation Hormonal therapy	13	9	
Chemotherapy	13	10	
Extent of Cancer			
Primary	13	10	0.677
Lymph node	12	12	0.838
**Skeletal Metastases**			
<6	0	2	-
6 - 20	7	4	0.546
>20	8	6	0.789
No skeletal Metastases	0	1	-
Median baseline PSA (ng/mL) (IQR)	221.5 (55.6 - 526.3)	282.4 (28.6 - 386.5)	0.629
Mean Cumulative activity in MBq (range)	23.5 (12 - 40)	30 (9 - 62.9)	0.205
The median number of ^225^Ac-PSMA-617 cycles (IQR)	2 (2 - 4)	3 (2.75 - 4)	0.200
Median follow-up duration in months (IQR)	9 (7 - 13)	10 (8 - 14)	0.766

IQR: Inter quartile range; SD: Standard deviation; PSA: Prostate specific antigen.

**Table 3:** Pre and post-therapy clinical response parameters

Variables	Pre-therapy	Post-therapy	P-value
VAS max	8 (0 - 10)	4.7 (0 - 9)	<0.0001
Analgesic score	3 (0 - 4)	2 (0 - 3)	0.001
KPS	60.4 (40 - 80)	75.5(40 - 90)	<0.0001
ECOG performance status	3 (1 - 4)	2 (0 - 4)	<0.0001

All values are mentioned in median and range. VAS: Visual analgesic score; KPS: Karnofsky Performance Status; ECOG: Eastern Cooperative Oncology Group.

**Table 4:** Overview of currently published literature on ^225^Ac-PSMA-617 TAT

Author	N	Treatment cycles (range)	Any PSA decline	>50% PSA fall	OS (months)	PFS (months)	CRR (%)	Administered Activity	ECOG Performance status
Kratochwil *et al.* 1	2	1 - 3	2	2	-	-	-	100 KBq/Kg	-
Kratochwil *et al.* 9	14	-	-	-	-	-	-	50 - 100 KBq/Kg	-
Kratochwil *et al.* 11	38	-	33/38 (87%)	24/38 (63%)	>12	7	5/38 (13%)	100 KBq/Kg	0/1 - 31 (80%) ≥2 - 7 (20%)
Sathekge *et al*. 12	17	11patients (2 - 3 cycles) 6 patients (>3 cycles)	-	14/17 (82%)	-	-	-	8-6 & 4 MBq	0/1 - 15 (88%) 2 - 2 (12%)
Sathekge *et al.* 19	73	-	83%	70 %	18 (16.2 -19.9)	15.2 (13.1 - 17.4)	21/73 (29%)	8 - 6 & 4 MBq	0/1 - 82% ≥2 - 18%
Khreish *et al.* 21	20	1 (tandem 225Ac +^177^Lu-PSMA-617	16/20 (80%)	13/20 (65%)	12 (1 - 23)	4.9 (3 - 6.5)		60 KBq/Kg (20 - 84)	0/1 - 12 (60%) 2 - 8 (40%)
Current Study	28	1 - 7	22/28 (78.6%)	11/28 (39%)	17 (16 - upper limit not reached)	12 (9 - 13)	2/22 (9%)	100 KBq/Kg	0/1 - 2 (7%) 2 - 6 (21%) 3 - 15 (54%) 4 - 5 (18%)

N: number of patients; PSA: Prostate specific antigen, OS: Overall survival, PFS: Progression-free survival, CRR: Complete response rate, KBq/Kg: Kilobecquerel per kilogram, MBq: Megabecquerel, ECOG performance status: Eastern Cooperative Oncology Group.

## Abbreviations

AA: abiraterone acetate
ADTs: androgen deprivation therapies
ALP: alkaline phosphatase
AS: analgesic score (AS)
BW: body weight
CI 95%: confidence interval
CR: complete response
CBC: complete blood counts
CTCAE v5.0: common toxicity criteria for adverse events a version 5.0
DCR: disease control rate
EBRT: external beam radiation therapy
ECOG: eastern cooperative oncology group
GFR: glomerular filtration rate
HR: hazard ratio
IQR: interquartile range
kBq: kilobecquerel
Kg: kilogram
KFT: kidney function tests
KPS: Karnofsky performance status
LFT: liver function tests
mCRPC: metastatic castration-resistant prostate cancer
MBq: megabecquerel
µCi: microcurie
MR: minimal response
OS: overall survival
PCWG3: prostate cancer working group criteria
PET/CT: positron emission tomography/computed tomography
PERCIST 1 criteria: PET Response Criteria in Solid Tumors
PD: progressive disease
PFS: progression-free survival
PR: partial response
PSA: prostate-specific antigen
PSMA: prostate specific membrane antigen
RLT: radioligand therapy
SD: stable disease
sPSA: serum prostate-specific antigen
TAT: targeted alpha therapy
VAS: visual analog score
