# Supplementary material for: Efficacy and safety of 225Ac-PSMA-617 targeted alpha therapy in metastatic castration-resistant Prostate Cancer patients
Source: Theranostics. 2020 Jul 23;10(20):9364–77. doi: 10.7150/thno.48107 (PMC7415797; doi:10.7150/thno.48107)
Supplement: Supplementary file 1 — Supplementary tables. [file thnov10p9364s1.pdf]

Supplementary Tables

Supplementary Table 1. Univariate and multivariate Cox proportional hazard regression overall survival analysis.

	Univariate Analysis			Multivariate Analysis	
Parameters	Median OS (months)	P (Log-rank)	HR; 95% CI	P	HR; 95% CI
Age (years)					
<71	17	0.771	1.261 (0.251 - 6.313)		
≥ 71	16				
Number of bone metastases					
≤ 20	17	0.980	0.981 (0.198 - 4.861)		
>20	16				
ECOG performance status					
0-2	6.8	0.067	6.823 (0.612 - 2.811)		
≥ 3					
ALP values (U/L)					
≤ 333	Not attained	0.588	1.512 (0.2 - 7.88)		
>333	17				
Any PSA Decline					
No	16	0.017	8.322 (1.595 - 43.516)		
Yes	Not attained				
PSA decline >50%					
No	16	0.022	6.611 (1.322 - 33.2)		
Yes	Not attained				
PSA progression >25%				0.033	12.2 (1.276 - 118)
Yes	8.5	0.0051	6.452 (0.616 - 7.87)		
No	17				
Number of ^{225}Ac-PSMA-617 cycles					
≤ 3 cycles	17	0.221	2.637 (0.524 - 13.2)		
>3 cycles	Not attained				
Previous chemotherapy					
Yes	17	0.484	0.486 (0.029 - 8.11)		
No	Not attained				
Previous ^{177}Lu-PSMA-617 therapy					
Yes	16	0.937	0.808 (0.138 - 4.735)		
No	17				
Concomitant therapies					
No	16	0.565	0.636 (0.1226 - 3.293)		
Yes	17				

OS: Overall survival; HR: Hazards ratio; 95% CI: 95% confidence interval; ECOG: Eastern cooperative oncology group performance status; ALP: Alkaline Phosphotase; PSA: Prostate specific antigen.

Supplementary Table 2. Univariate and multivariate Cox proportional hazard regression progression-free survival analysis.

	Univariate Analysis			Multivariate Analysis	
Parameters	Median PFS (months)	P (Log-rank)	HR; 95% CI	P	HR; 95% CI
Age (years)					
<71	10	0.696	0.780 (0.211 – 2.882)		
≥ 71	12				
Number of bone metastases					
≤ 20	Not attained	0.129	0.325 (0.0879 - 1.286)		
>20	8				
ECOG performance status					
0-2	6	0.067	6.834 (0.612 - 12.811)		
≥ 3					
ALP values (U/L)					
≤ 333	12	0.938	0.951 (0.240 - 3.760)		
>333	10				
Any PSA Decline				0.002	11.2 (2.333 – 54.632)
No	7	0.0001	10.722 (2.361 - 49.293)		
Yes	Not attained				
PSA decline >50%					
No	8	0.019	7.414 (2.005 - 27.430)		
Yes	Not attained				
PSA progression >25%					
Yes	7	0.0001	6.763 (1.177 - 40.943)		
No	Not attained				
Number of ^{225}Ac-PSMA-617 cycles					
≤ 3 cycles	8	0.221	2.199 (0.594 - 8.136)		
>3 cycles	12				
Previous chemotherapy					
Yes	12	0.919	0.905 (0.104 - 7.865)		
No	Not attained				
Previous ^{177}Lu-PSMA-617 therapy					
Yes	10	0.194	1.892 (0.481 - 7.440)		
No	12				
Concomitant therapies					
No	12	0.479	1.562 (0.392 - 6.215)		
Yes	Not attained				

PFS: Progression-free survival; HR: Hazards ratio; 95% CI: 95% confidence interval; ECOG: Eastern cooperative oncology group performance status; ALP: Alkaline Phosphotase; PSA: Prostate specific antigen.

Supplementary Table 3. Adverse events according to CTCAE v5.0.

Baseline							After radioligand therapy				
Event	Grade 0	Grade 1	Grade 2	Grade 3	Grade 4		Grade 0	Grade 1	Grade 2	Grade 3	Grade 4
Hemoglobin	2	19	7	0	0		0	15	12	1	0
Platelet counts	26	2	0	0	0		24	2	2	0	0
WBCs	24	4	0	0	0		26	9	2	0	0
Creatinine	26	2	0	0	0		24	4	0	0	0
Xerostomia	0	0	0	0	0		20	3	5	0	0

WBC: White blood cell counts; CTCAE: Common toxicity criteria for adverse events, ALP: Alkaline phosphatase.
